# High Sensitivity to Interpersonal Interaction: Development of a Measurement

**DOI:** 10.5334/pb.1267

**Published:** 2024-12-31

**Authors:** Karina Salud Montoya-Pérez, Ferran Padrós-Blázquez, Rocío Montoya-Pérez

**Affiliations:** 1 Facultad de Psicología, Universidad Michoacana de San Nicolás de Hidalgo, Michoacán, México; 2 Instituto de Investigaciones Químico-Biológicas, Universidad Michoacana de San Nicolás de Hidalgo, Michoacán, México

**Keywords:** adults, high interpersonal sensitivity, measurement, sensory processing sensitivity

## Abstract

Interpersonal sensitivity is an aspect of Sensory Processing Sensitivity (SPS) that has been unexplored precisely despite potentially playing an even more significant role in individuals with SPS. The results of various studies on individuals with SPS suggest that this trait is accompanied by a high interpersonal sensitivity, which refers to an increased sensitivity to the emotional states of individuals with whom one interacts; however, no measurement instrument directly evaluates it. This research aimed to develop an instrument to assess high interpersonal sensitivity and analyze its psychometric properties. Four hundred twenty-nine university students aged 18 to 29 participated. Confirmatory Factor Analysis supported a three-factor structure (awareness of subtleties, overstimulation, and persistent effect) consistent with Exploratory Factor Analysis findings. The internal consistency values for the total scale and the three factors were adequate, and the validity evidence was congruent with the construct. Despite needing further studies, the High Interpersonal Sensitivity Scale (HISS) shows adequate psychometric properties for measuring high interpersonal sensitivity in adults.

## Introduction

Individual differences exist in the experience of interacting with others. The speed and intensity of reaction, the amount of information perceived, and the way information is processed are influenced by the degree of sensitivity one possesses.

Building upon the studies of Aron & Aron (1997), it is proposed that the ability to detect and respond to internal and external stimuli, including interactions with others, may be particularly pronounced in individuals with Sensory Processing Sensitivity (SPS). This temperament trait is characterized by 1) deep cognitive processing of stimuli, 2) heightened emotional reactivity, 3) vulnerability to sensory overstimulation, and 4) heightened awareness of subtleties in the environment, including people’s emotional states (Acevedo et al., 2014; Aron et al., 1997; Aron et al., 2012; Jagiellowicz et al., 2020).

In detail, fMRI studies on individuals with SPS (e.g., Acevedo et al., 2014; Acevedo et al., 2017) demonstrate that this trait is associated with deep processing and heightened responsiveness to emotional signals from others. These studies highlight increased activation in brain regions associated with reward processing, memory, emotion, empathy, and awareness.

For this reason, the role of the parenting environment in the development of emotional regulation strategies in individuals with SPS has also been studied. Specifically, it is reported that adverse environments, poor parenting, or lack of social support have a worse impact on children with SPS (Boterberg & Warreyn, 2016; Liss et al., 2008), often resulting in depression and anxiety problems in adulthood (e.g., Bakker & Moulding, 2012). Conversely, environments with low levels of stress and adequate emotional support promote greater creativity (Bridges & Schendan, 2019), improved social skills (Slagt et al., 2018), and more effective emotional regulation strategies (Brindle et al., 2015).

As noted, interpersonal interaction, especially in the early years, plays a more crucial role in individuals with SPS than those without this trait, largely influencing their well-being or psychological development. Furthermore, SPS characteristics such as heightened awareness of subtleties and emotional states of others and intense emotional experiences suggest that this trait is also accompanied by high interpersonal sensitivity. This characteristic refers to an increased sensitivity to the emotional states of individuals with whom one interacts.

But unlike the emotional reactivity proposed by Davis (1980), which involves the activation of various cognitive and emotional processes to understand the emotional state of others, as well as adopt an appropriate perspective and respond correctly, high interpersonal sensitivity refers to enhanced responsiveness to the perception of the emotional components of interaction with others (input) rather than an emotional and/or cognitive reaction as a result (output).

On the other hand, the most used definitions regarding interpersonal sensitivity are: “the ability to feel, perceive accurately, and respond appropriately to one’s personal, interpersonal, and social environment” (p. 3) (Bernieri, 2001) and “an undue and excessive awareness of, and sensitivity to, the behavior and feelings of others” (p. 342) (Boyce & Parker, 1989). Both refer to observing behaviors that also occur in people with SPS. However, they are insufficient to describe high interpersonal sensitivity because they are based on a specific and distinct theoretical foundation. In other words, neither definition likely considers the existence of the trait of SPS with its innate condition. Bernieri (2001) refers to an acquired skill or capacity. Although Boyce & Parker (1989) describe characteristics related to SPS, they are likely those observable in the presence of psychopathology.

Additionally, the literature search yielded no studies explicitly exploring the relationship between high interpersonal sensitivity and SPS. It did not reveal a specific reference within the conceptual framework of SPS as a distinctive feature within the construct’s definition. However, the results reported in studies on SPS and its relationship with caregiving environments highlight the need to understand how high interpersonal sensitivity seems to enhance the beneficial or harmful effects of interactions with others from the earliest years of life. Therefore, establishing the theoretical and empirical link between high interpersonal sensitivity and SPS is a necessary and pertinent contribution.

It is worth noting that scales designed to assess interpersonal sensitivity, such as the Perceptual Decoding Ability Test (PDA) (Zuckerman & Larrance, 1979), the Test of Sensitivity to Social Interactions (TESIS) (Barraca et al., 2009), or the Interpersonal Sensitivity Test (Boyce & Parker, 1989), are instruments that evaluate an adaptive social ability or, in the case of the latter, a maladaptive personality trait.

Regarding the SPS, it is valid to assume that since it is a temperament trait that appears to be innate, high interpersonal sensitivity does not either correspond to an acquired capacity or skill, or an alteration or malfunction but rather to an inherent characteristic. Therefore, assessing interpersonal sensitivity in individuals with SPS should focus on identifying the effect that interactions with other people can have on their emotional and psychological functioning, and the mentioned instruments were not designed with that objective.

In the same way, the most widely used scale in studies on SPS, the Highly Sensitive Person Scale (HSPS) (Aron & Aron, 1997), does not include a dimension or subscale specifically investigating interpersonal sensitivity. This instrument only features two items related to interaction with others: *Do other people’s moods affect you? When people feel uncomfortable in a physical space, do you usually identify or know what needs to be done to make them feel more comfortable? (e.g., changing the light or the seating arrangement)*. Though it considers SPS characteristics like emotional reactivity, awareness of subtleties, and empathetic responsiveness, it does not explore interpersonal interaction and its effects with depth.

Recently, the Sensory Processing Sensitivity Questionnaire (SPSQ) (De Guch et al., 2022) was developed to incorporate the positive aspects of the SPS that the HSPS failed to include, as noted by some studies (e.g., Greven et al., 2019; Montoya-Pérez et al., 2021; Montoya-Pérez & Padrós-Blázquez, 2024). To do this, items from both the HSPS and the Adult Temperament Questionnaire (ATQ) were taken (Evans & Rothbart, 2007). The social and affective dimensions included a social-affective sensitivity and an understood associative sensitivity. This dimension is made up of seven items. Through statements such as: *Sometimes I notice sad eyes hidden by a smile*; *I’m usually surprised when a person’s tone of voice doesn’t match their words*, or; *When people are uncomfortable, I know how to calm them down*, it seems focused primarily on recognition of emotional states and heightened awareness of subtleties. These characteristics of the SPS are fundamental to understanding the effect of interactions with other people. Still, it is necessary to consider different elements of interest that have not been sufficiently explored in this sense, such as overstimulation and intense emotional experience.

The aim of this research is to develop and validate an instrument for assessing the presence of high interpersonal sensitivity in adults.

## Method

### Item Writing and Expert Review Phase

Based on in-depth interviews with 20 adults identified with SPS, 45 statements were drafted related to their emotional experiences in interactions with others since early childhood. Participants were asked to reflect on experiences that were pleasant, unpleasant, significant, insignificant, memorable, emotionally impactful, or that affected their way of relating to others.

It should be noted that since cutoff scores for the scale used to assess SPS presence have not been defined, low, medium, and high levels were determined using total scores obtained from the HSPS (Highly Sensitive Person Scale) (minimum, maximum, first and third quartiles, mean, and median). The defined levels were low, 22 to 62 points; medium, 63 to 88 points; and high, 89 to 114.

The statements resulting from the interviews were grouped considering their potential similarity to SPS characteristics outlined in the Introduction section as part of its definition (Acevedo et al., 2014; Aron et al., 1997; Aron et al., 2012; Jagiellowicz et al., 2020). They were categorized as follows: 1) *awareness of subtleties*, referring to heightened awareness of subtle environmental cues, including the emotional states of others; 2) *emotional reactivity*, based on increased emotional responsiveness; 3) *empathic response*, involving greater empathy towards others, and; 4) *overstimulation*, centered on vulnerability to sensory overstimulation.

It is important to note that a set of statements was crafted based on a common theme observed across all interviewees: a prolonged emotional effect accompanying interpersonal interactions where intense, mostly unpleasant emotions were experienced. Although this specific reference was not found in the reviewed empirical evidence related to SPS, it was also decided to include it, termed *persistent effect*.

Within the 45 drafted statements, it was noted that some expressed similar ideas differently. Therefore, a decision was made to select clearer and easier to understand. Additionally, there were discarded statements describing characteristics such as gratitude, responsibility, or commitment.

Once a version of 25 items (five dimensions with five items each) was finalized, it underwent content validity assessment by five expert judges. They reviewed each statement’s wording and syntax to ensure each item corresponded accurately to its designated dimension or characteristic. The 25 proposed items were retained because they achieved Aiken’s V and Kendall’s W scores above 0.80, indicating high agreement regarding relevance and clarity.

### Empirical Phase

#### Participants

Using convenience sampling, 429 university students from various educational programs participated, excluding psychology, to avoid potential bias in the results. The sample consisted of 250 (58.27%) women, 172 (40.09%) men, and 7 (1.63%) individuals of a different gender, aged between 18 and 29 years, with a mean age of 20.41 and an *SD* of 1.91. To participate in the study, subjects met the following inclusion criteria: (a) be over 18 years of age and (b) sign an informed consent form agreeing to participate voluntarily in the research.

#### Instruments

High Interpersonal Sensitivity Scale (HISS). This self-report instrument was developed for the present research to assess levels of high interpersonal sensitivity in adults. It consists of 25 items evenly distributed across five dimensions: 1) *awareness of subtleties*, 2) *emotional reactivity*, 3) *emphatic response*, 4) *overstimulation*, and 5) *persistent effect*. For each statement, respondents are asked to indicate on a 4-point Likert scale the extent to which the statement describes them. The response options are: 1 = not at all, 2 = slightly, 3 = moderately, and 4 = completely.

**Highly Sensitive Person Scale (HSPS)** (Aron & Aron, 1997). Translated and adapted for the Mexican population (Montoya-Pérez et al., 2019). It is a self-report scale designed to measure the degree of sensitivity in adults. It consists of 17 items with Likert-type responses ranging from 1 (Not at all) to 7 (Extremely), which are answered based on the person’s feelings. All items are scored in the same direction, so higher scores indicate higher sensitivity. Principal component analysis suggested a two-factor solution that explained 30% of the variance: 1) *processed sensitivity* (PS) with 13 items, and 2) *low sensory threshold* (LST) with 4 items. Reliability analysis reported an α coefficient of 0.89.

**Interpersonal Reactivity Index (IRI)** (Davis, 1980).Translated and adapted for the Mexican population (Ahuatzin-González et al., 2019). It is a self-report scale with 28 items designed to assess empathy in a multidimensional manner. It comprises a cognitive component with two dimensions, *perspective taking* and *fantasy*, and an affective component with two dimensions: *empathic concern* and *personal distress*. These two components are distributed across four subscales with 7 items each. Each dimension has 7 items, and responses are given on a Likert scale with five response options, where higher scores indicate a higher presence of the measured dimension. The reported reliability for the total scale was α = 0.81.

### Procedure

Once authorized by the directors and professors of Schools and Faculties at a public university, students were invited to participate in the study during class time. Those who signed the informed consent form completed the HSPS, IRI, and HISS instruments. The administration of the scales took approximately 25 minutes.

### Ethical Considerations

Before conducting the research, the project was reviewed and approved by the Faculty of Psychology ethics committee at the Universidad Michoacana de San Nicolás de Hidalgo. Participation was voluntary and ensured by signing an informed consent form that guaranteed the confidentiality and anonymity of personal data.

### Data Analysis

Data analysis was conducted using R statistical software version 4.0.2 (R Core Team, 2020). Exploratory Factor Analysis (EFA) was performed using the *psych* package (Revelle, 2019), and Confirmatory Factor Analysis (CFA) was conducted using the *lavaan* package (Rosseel, 2012). Internal consistency (Cronbach’s alpha and McDonald’s omega) was assessed using JASP software version 0.8.5.1 (Jasp Team, 2023).

#### Exploratory Factor Analysis

The total sample was randomly split into two parts. Exploratory Factor Analysis (EFA) was conducted on the first half of the sample, with Confirmatory Factor Analysis (CFA) reserved for the second half.

Before conducting the Exploratory Factor Analysis (EFA), Kaiser-Meyer-Olkin (KMO) and Bartlett’s test of sphericity were performed as measures of the adequacy of the data for factor analysis. A KMO value above 0.5 and a significance level below 0.05 for Bartlett’s test were set as criteria.

The EFA was conducted using the principal axis factoring method with varimax rotation. Criteria for retaining items included factor loading greater than 0.45 on a single factor, factor loadings not exceeding 0.30 on other factors, and item content congruence with the factor.

#### Confirmatory Factor Analysis

CFA was conducted using the maximum likelihood method. Model fit was evaluated using various indices. Absolute fit indices included χ^2^ and the standardized root mean square residual (SRMR). Comparative fit indices included the Comparative Fit Index (CFI), Tucker-Lewis Index (TLI), and Akaike Information Criterion (AIC). The Root Mean Square Error of Approximation (RMSEA) was also used to assess model parsimony (Moral, 2006).

For the χ^2^ test, a non-significant result indicates a good fit. CFI and TLI values should exceed 0.90 (higher values indicate better fit). SRMR values below .08 are considered acceptable, with lower values indicating better fit. RMSEA values should be below .08 for acceptable fit or close to .05 for good fit (Browner & Crudeck, 1993; Byrne, 2001). AIC, an unbounded selection criterion, compares models fitted to the same data; smaller values indicate a better fit.

#### Reliability

Reliability was estimated using Cronbach’s alpha and McDonald’s Omega coefficients, with a confidence level of 95%. Values above 0.7 were considered acceptable (Campo-Arias & Oviedo, 2008).

#### Convergent Validity

Convergent validity was assessed through correlation analysis, evaluating the relationship between the HISS’s total scores and its factors and the HSPS’s and IRI’s total scores and factors.

## Results

### Exploratory Factor Analysis

The KMO index (0.85) and Bartlett’s test (p < 0.001) indicated appropriate values for conducting exploratory factor analysis (EFA). The principal axis factoring method with varimax rotation was used for the 25 items initially included in the HISS. Firstly, five dimensions were obtained based on criteria of eigenvalues greater than 1. However, considering the factor loadings of each item, their theoretical coherence, and their grouping within factors, 11 items describing dimensions of *emotional reactivity* and *empathetic response* were discarded.

With the remaining 14 items, EFA revealed a three-factor solution using the same extraction method and rotation. These items exhibited loadings above 0.45 in their respective factors, collectively explaining 47% of the total variance (see Table 1). The three derived dimensions from the analysis were: factor 1, *awareness of subtleties*, explaining 19% of the variance with 5 items; factor 2, *overstimulation*, explaining 15% of the variance with 5 items; and factor 3, *persistent effect*, explaining 13% of the variance with 4 items.

**Table 1:** Final Exploratory Factor Analysis.


ITEM	FACTOR SATURATION		

	AS	OS	PE

HISS 1	0.72		

HISS 6	0.64		

HISS 11	0.63		

HISS 16	0.61		

HISS 21	0.61		

HISS 24		0.84	

HISS 14		0.74	

HISS 9		0.73	

HISS 4		0.67	

HISS 19		0.54	

HISS 20			0.71

HISS 10			0.64

HISS 15			0.54

HISS 25			0.53


*Note:* AS = awareness of subtleties; OS = overstimulation; PE = persistent effect.

### Confirmatory Factor Analysis

Confirmatory factor analysis compared four different models:

1. The model was derived from EFA and included 14 items and three factors (*awareness of subtleties, overstimulation*, and *persistent effect*).
2. A single-factor model with the 14 items resulting from EFA.
3. The model has an initial 25 items and five factors (*awareness of subtleties, emotional reactivity, empathetic response, overstimulation*, and *persistent effect*).
4. A single-factor model with the initial 25 items.

The findings are presented in Table 2.

**Table 2:** CFA goodness-of-fit indices (four models).


MODEL	χ*^2^*	χ*^2^*/*gl*	*P*	AIC	CFI	TLI	SRMR	RMSEA

1	125.152	1.69	<.001	7062.40	0.94	0.93	0.06	0.06

2	553.24	7.18	<.001	7484.48	0.45	0.35	0.17	0.17

3	508.50	1.92	<.001	12355.84	0.85	0.83	0.09	0.07

4	1064.68	3.87	<.001	12892.02	0.51	0.47	0.12	0.12


Regarding the χ^2^ test, it is important to note that none of the models showed a satisfactory result, as this test should be non-significant to confirm a good fit. However, Littlewood and Bernal (2011) point out that χ^2^ is prone to error when applied to large samples, i.e., more than 200 observations. Therefore, other indices are recommended for model confirmation. Models 1, 3, and 4 exhibit a χ^2^/df value <5, suggesting an acceptable fit (Bollen, 1989).

**Figure 1:**
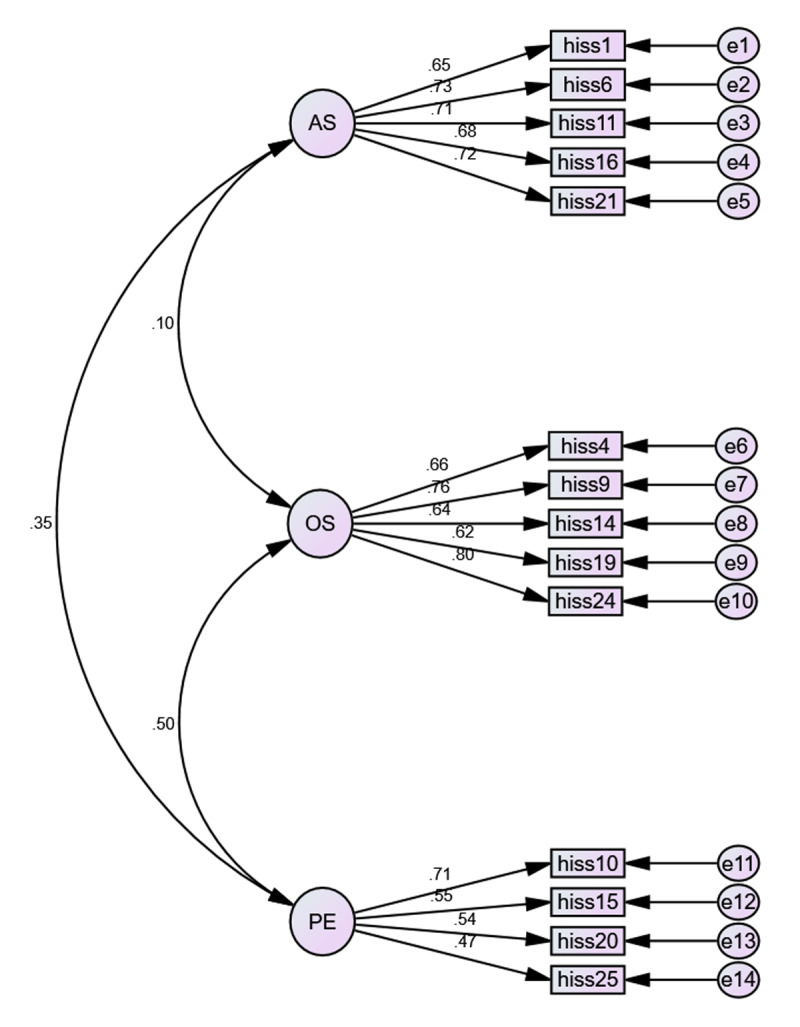
Model 1 (14 items y 3 factors). AS = awareness of subtleties; OS = overstimulation; PE = persistent effect.

Additionally, CFI and TLI indices were adequate for models 1 and 3. Concerning SRMR and RMSEA, the best values are reported for models 1 and 3. However, AIC confirms that model 1 (derived from exploratory factor analysis) demonstrates the best parsimony. Therefore, the model derived from EFA with 14 items and three factors (*awareness of subtleties, overstimulation*, and *persistent effect*) shows the best fit (see Figure 1).

#### Reliability and Factor Correlations

Internal consistency for the total HISS score and each factor was assessed using Cronbach’s alpha and McDonald’s omega coefficients (see Table 3). The total HISS score yielded α = 0.803 and ω = 0.804. Factor 1, *awareness of subtleties*, showed the highest indices, similar to those obtained for the total score. The other factors (*overstimulation* and *persistent effect*) demonstrated values above 0.6 for Cronbach’s alpha and close to 0.7 for McDonald’s omega, which are considered acceptable (see Table 3).

**Table 3:** Internal consistency of the HISS and correlations between the factors.


	INTERNAL CONSISTENCY		CORRELATIONS			
	
FACTORS	CRONBACH’S α	MCDONALD’S ω	AS	OS	PE	TOTAL

AS	0.803	0.804	–	**0.15**	**0.21**	**0.61**

OS	0.618	0.692		–	**0.36**	**0.76**

PE	0.691	0.693			–	**0.73**


*Note:* AS = awareness of subtleties; OS = overstimulation; PE = persistent effect. Statistically significant correlations were obtained in bold (p < 0.01 and p < 0.001).

The results regarding the relationship between factors indicated that their correlations were significant but low. Each factor also showed a significant, moderate correlation with the total scale score.

#### Convergent Validity

As evidence of convergent validity, a correlation analysis was conducted between the final version of HISS and its factors with HSPS and IRI scores and their respective dimensions. As observed in Table 4, only the correlations between the *overstimulation* dimension (HISS) and *empathic concern* (IRI) were not statistically significant. The results of correlations between the total scores of the scales show moderate correlations, with the highest correlation observed between HISS and HSPS, followed by the correlation between HSPS and IRI (see Table 4).

**Table 4:** Correlations between the scales and their factors.


	HSPS	PS	LST	IRI	PT	FS	EC	PD

HISS	**0.64**	**0.60**	**0.57**	**0.50**	**0.30**	**0.33**	**0.38**	**0.49**

AS	**0.23**	**0.21**	**0.22**	**0.28**	**0.27**	**0.16**	**0.28**	0.09

OS	**0.55**	**0.49**	**0.53**	**0.26**	**0.12**	**0.20**	0.09	**0.40**

PE	**0.60**	**0.60**	**0.42**	**0.59**	**0.29**	**0.36**	**0.51**	**0.56**

HSPS	–	**0.97**	**0.80**	**0.55**	**0.24**	**0.36**	**0.36**	**0.66**

PS		–	**0.63**	**0.56**	**0.23**	**0.36**	**0.38**	**0.67**

LST			–	**0.39**	**0.21**	**0.27**	**0.20**	**0.46**

IRI				–	**0.70**	**0.71**	**0.78**	**0.71**

PT					–	**0.20**	**0.50**	**0.23**

FS						–	**0.28**	**0.54**

EC							–	**0.39**

PD								–


*Note:* HISS = total score; AS = awareness of subtleties; OS = overstimulation; PE = persistent effect; HSPS = total score; PS = processed sensitivity; LST = low stimulation threshold; IRI = total score; PT = perspective taking; FS = fantasy; EC = emphatic concern; PD = personal distress. Statistically significant correlations were obtained in bold (p < 0.01 and p < 0.001).

The results showed weak and moderate correlations between the HISS dimensions and other variables. Weak correlations were observed between the *awareness of subtleties* dimension and the rest of the variables (HSPS and its factors, IRI and its factors), as well as between the *overstimulation* dimension and IRI and its dimensions, except for the *personal distress* dimension, which showed a moderate correlation.

Moderate correlations were also found between the *overstimulation* dimension and HSPS and its factors. The *persistent effect* dimension exhibited moderate correlations with the rest of the variables (HSPS and its factors, IRI and its factors), except for the IRI dimensions of *perspective taking* and *fantasy*, which showed weak correlations (see Table 4).

Finally, concerning the descriptive data, it should be noted that factor scores were derived from the sum of item values divided by the number of items in the factor, and the total score was obtained from the sum of the scores of the three factors. The data are presented in Table 5. Factor and total scale scores cover practically all possible scores, and the skewness and kurtosis values for all scores indicate a normal distribution.

**Table 5:** Description of the percentiles of the three factors and the total score of the HISS.


PERCENTILE	AS	OS	PE	HISS

1	1.46	1.00	1.25	4.86

10	2.00	1.60	2.25	6.85

20	2.40	1.80	2.75	7.60

25	2.60	2.00	2.75	7.90

30	2.60	2.20	3.00	8.20

40	2.80	2.40	3.25	8.60

50	3.00	2.60	3.25	9.00

60	3.20	2.80	3.50	9.35

70	3.40	3.00	3.75	9.65

75	3.60	3.20	3.75	9.80

80	3.60	3.40	3.75	10.05

90	3.80	3.80	4.00	10.80

99	4.00	4.00	4.00	11.80

**Medium**	3.01	2.62	3.23	8.86

**SD**	0.64	0.79	0.65	1.47

**Min**	1.20	1.00	1.00	3.85

**Max**	4.00	4.00	4.00	12.00

**Skewness**	–0.407	0.016	–0.872	–0.355

**Kurtosis**	–0.541	–0.864	0.266	0.047


*Note:* HISS = total score; AS = awareness of subtleties; OS = overstimulation; PE = persistent effect; SD = standard deviation.

## Discussion

The main objective of this research was to develop an instrument to assess high interpersonal sensitivity. It has been noted that individuals with Sensory Processing Sensitivity (SPS) are also more sensitive in their interactions with others (e.g., Jagiellowicz et al., 2020), especially in parenting contexts (Booth et al., 2015; Slagt et al., 2018). However, an instrument specifically investigating this aspect of SPS still needs to be developed, hence the relevance of this research.

The goodness-of-fit indices obtained in the initial exploratory factor analysis and the distribution of items across factors provided decisive elements for excluding 11 items from the initially proposed version, which led to the omission of two of the first considered dimensions: *emotional reactivity* and *empathetic response*.

The final structure of the HISS retains three of the five initially considered dimensions: *awareness of subtleties, overstimulation*, and *persistent effect*. The reliability index of the *awareness of subtleties* factor is adequate. However, the reliability of the other two factors falls just short of being acceptable; nevertheless, some authors consider values above six and a half acceptable for the McDonald’s omega coefficient (e.g., Ventura-León & Caycho-Rodríguez, 2017). The low reliability of these factors could be attributed to the small number of items in each factor.

The correlation between the factors was significant but low; however, each factor’s correlation with the total scale was moderate. These results suggest that these dimensions are independent but contribute to a larger construct.

Specifically, the three dimensions comprising the final internal structure of the HISS capture distinctive characteristics of SPS that describe the process of exposure to stimuli in individuals with high interpersonal sensitivity. Increased *awareness of subtleties* can lead to *overstimulation* of the nervous system when exposed for prolonged periods, thereby producing a *persistent* emotional *effect* from the experience.

The results of the exploratory factor analysis suggest that the items designed to capture dimensions of *emotional reactivity* and *empathic response* are grouped into a single factor. This may indicate a need for more specificity in item wording and potential shared content between these two dimensions. Given that these aspects of SPS have primarily been studied using methods such as fMRI, it is crucial to explore them with more precise and differentiated assessments, considering they refer to heterogeneous constructs.

Similarly, these results reinforce the proposal that high interpersonal sensitivity describes an increased responsiveness to the perception of emotional components present in interactions with people rather than an emotional response derived from this perception. Therefore, since it is not an empathic or emotional reactivity, it can explain why the *empathic response* and *emotional reactivity* dimensions were not part of the HISS.

Regarding convergent validity, the moderate correlations between the HISS and HSPS and between the HISS and IRI indicate a relationship but no overlap. This pattern is also reflected in the correlations between the factors of the three scales, where only certain factors showed moderate correlations, most of which were weak values.

In particular, the moderate correlation between the dimension of *overstimulation* in the HISS and the HSPS with its factors aligns with expectations based on findings reported by Montoya-Pérez et al. (2021). Their study revealed a moderate correlation between the total score of the HSPS and the DESR-E, an instrument designed to assess difficulties in emotional regulation. Consequently, they suggested a potential bias in the HSPS structure towards problems associated with SPS. The results of the current study appear to confirm this hypothesis, especially considering that the dimension of *personal distress* in the IRI also moderately correlated with the HSPS and its factors, similar to the *overstimulation* dimension in the HISS.

Moreover, the moderate correlation between the dimension of *persistent effect* in the HISS and the HSPS with its factors and with the IRI and its *empathic concern* and *personal distress* factors strengthens the notion that SPS amplifies emotional experience. High interpersonal sensitivity can lead to both adaptive and comforting social experiences and overwhelming and demanding experiences, depending on the quality of interactions and the context in which they occur.

Similarly, these moderate correlations between the HSPS and the HISS support the notion that high interpersonal sensitivity can be considered an integral aspect of the SPS trait. The current research findings suggest that a comprehensive assessment of SPS should consider sensitivity to physical stimuli and sensitivity in interpersonal relationships. Furthermore, the positive relationship between interpersonal sensitivity and empathy, as measured by the IRI, supports the idea that the SPS trait could confer an evolutionary advantage under favorable environmental conditions (such as parenting).

Within the limitations of this study, one notable aspect is the lack of control over variables related to SPS, such as social anxiety (Hofmann & Bitran, 2007), social skills (Hilton et al., 2007), and emotional intelligence (O’Neill, 2023), which could enhance the evidence of validity. Additionally, test-retest reliability has yet to be investigated. It would be beneficial for future studies to explore the relationship between the scale and these variables and assess test-retest reliability.

Future studies should also examine the HISS’s discriminant validity and establish an optimal cutoff point based on its sensitivity and specificity for distinguishing between individuals with high personal sensitivity traits and those without. A standard based on classification through in-depth interviews conducted by expert evaluators would be necessary.

Another significant limitation is that the sample comprised only young people and university students. Therefore, the results cannot be generalized to older populations or those with lower levels of education. Future research should consider expanding the age range and educational levels to increase the representativeness of the findings.

Finally, the results of this study indicate that the HISS (High Interpersonal Sensitivity Scale) has adequate psychometric properties to discriminate in adults the presence of high interpersonal sensitivity, a distinctive characteristic of HSP expressed through heightened sensitivity in interactions with others.

## Supplementary data

HISS (High Interpersonal Sensitivity Scale) and supplementary data to this article can be request to the corresponding author.
